# LettuceTrack: Detection and tracking of lettuce for robotic precision spray in agriculture

**DOI:** 10.3389/fpls.2022.1003243

**Published:** 2022-09-30

**Authors:** Nan Hu, Daobilige Su, Shuo Wang, Purevdorj Nyamsuren, Yongliang Qiao, Yu Jiang, Yu Cai

**Affiliations:** ^1^College of Engineering, China Agricultural University, Beijing, China; ^2^School of Mechanical Engineering and Transportation, Mongolian University of Science and Technology, Ulaanbaatar, Mongolia; ^3^Australian Centre for Field Robotics (ACFR), The University of Sydney, Sydney, NSW, Australia; ^4^Horticulture Section, School of Integrative Plant Science, Cornell University, Geneva, NY, United States

**Keywords:** agriculture, detection, tracking, MOT, deep learning, precision spray, robotics

## Abstract

The precision spray of liquid fertilizer and pesticide to plants is an important task for agricultural robots in precision agriculture. By reducing the amount of chemicals being sprayed, it brings in a more economic and eco-friendly solution compared to conventional non-discriminated spray. The prerequisite of precision spray is to detect and track each plant. Conventional detection or segmentation methods detect all plants in the image captured under the robotic platform, without knowing the ID of the plant. To spray pesticides to each plant exactly once, tracking of every plant is needed in addition to detection. In this paper, we present LettuceTrack, a novel Multiple Object Tracking (MOT) method to simultaneously detect and track lettuces. When the ID of each plant is obtained from the tracking method, the robot knows whether a plant has been sprayed before therefore it will only spray the plant that has not been sprayed. The proposed method adopts YOLO-V5 for detection of the lettuces, and a novel plant feature extraction and data association algorithms are introduced to effectively track all plants. The proposed method can recover the ID of a plant even if the plant moves out of the field of view of camera before, for which existing Multiple Object Tracking (MOT) methods usually fail and assign a new plant ID. Experiments are conducted to show the effectiveness of the proposed method, and a comparison with four state-of-the-art Multiple Object Tracking (MOT) methods is shown to prove the superior performance of the proposed method in the lettuce tracking application and its limitations. Though the proposed method is tested with lettuce, it can be potentially applied to other vegetables such as broccoli or sugar beat.

## 1. Introduction

Robotic application in precision agriculture has become a popular topic recently. Deploying robots in agricultural applications has the potential to significantly reduce the labor cost of repetitive tasks such as weeding (Lee et al., 2014; McCool et al., 2018; Jiang et al., 2020), fruit detection and yield estimation (Bargoti and Underwood, 2017), harvesting (Bac et al., 2017; Kurita et al., 2017; Sa et al., 2017), fertilizer or pesticide application (Adamides et al., 2017), crop mapping (Dong et al., 2017), and plant phenotyping (Ruckelshausen et al., 2009). In the case of robotic fertilizer and pesticide application in lettuce farms, compared to the conventional agricultural standard of treating the land indiscriminately, robotic autonomous spraying allows the crop to be targeted individually (Chebrolu et al., 2017). Not only does this make spraying more economical, it is also more eco-friendly. To precisely spray individual plants only once, the perception system of the robot needs to be able to detect crops against soil and weeds, as well as identify and track all individual crops.

There have been plenty of studies that exist in the literature, which successfully resolved the detection of individual plants of vegetables (Saleem et al., 2021; Jin et al., 2022; Ulloa et al., 2022). They allow robots to use their vision sensors to capture images of the farm field, and find the locations of plants in the images. With the detection results, the robot can spray each plant in the captured image precisely. However, only with detection results, the robot is unable to know which plants it has sprayed already when it travels through the farm lanes, without tracking each individual plant. To spray each plant exactly once, existing methods for robotic precision spray usually require the robot to travel in one direction and at a fixed distance to make sure the images continuously captured by the robot exactly follow one another and without the same plant in two images. When the robot needs to stop or slightly reverse back for obstacle avoidance, human intervention is needed to prevent the same plant to be sprayed twice, making the autonomy of the robot reduced significantly. Another common approach to tackle this problem is to use RTK-GPS or Simultaneous Localization and Mapping (SLAM) techniques to record the geometric locations of plants. However, usage of accurate RTK-GPS increases the cost of the robot considerably, and it also does not work in a greenhouse environment. Vision based SLAM techniques are not always robust, especially in the farm environment, and failure of them will directly lead to failure of spray action.

**Figure 1:**
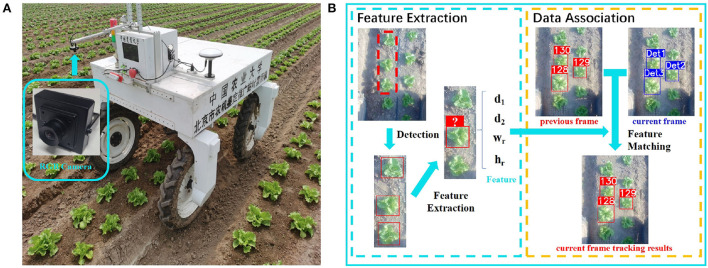
Overview of the proposed method. **(A)** VegeBot: The agricultural robot designed by the China Agricultural University, which travels through a lettuce farm, detects and tracks each plant, and sprays them precisely. **(B)** The proposed method detects lettuces and extracts features of the targets. Take the target in the red dotted box in the figure as an example, a novel feature for the middle target is obtained with the help of the upper and lower targets to reveal its identity information. The novel matching approach proposed in the paper can successfully re-identify the same target even if it disappears from the camera's field of view for a long time and re-appears again.

In this paper, we present LettuceTrack, a perception pipeline that incorporates the detection and tracking of lettuces using a camera attached to an agricultural robotic platform. As shown in Figure 1, a RGB camera is fixed facing downward in front of the VegeBot, an agricultural robot designed by the China Agricultural University, which is used to detect and track each plant when the robot travels through the lettuce farm. The proposed method detects lettuces and forms location features of them. Take the target in the red dotted box in the figure as an example, a novel feature for the middle target is obtained with the help of the upper and lower targets to reveal its identity information. It is combined with the novel matching approach proposed in the paper to successfully re-identify the same target even if it disappears from the camera field of view for a long time and re-appears again. The details of the feature extraction and the matching method are given in Section 3.2.

The contributions of the paper are 2-fold. First, we proposed a deep learning-based Multiple Object Tracking (MOT) method to solve the joint detection and tracking of lettuces problem for agricultural robots to perform the precision spray task. With tracking incorporated, the robot relieves the requirement of traveling in one direction and at a fixed distance, so it can stop or reverse back whenever it needs. Second, we introduced a novel feature to help identify each individual plant, which makes it possible for the robot to successfully re-identify the same plant even when it reverses back and sees the plant that has been seen by the robot and gone out of sight before, where conventional Multiple Object Tracking (MOT) methods usually fail. Experimental results have been conducted to show the effectiveness of the proposed method, and a comparison with four state-of-the-art MOT methods is provided to prove the superior performance of the proposed method in the lettuce tracking application and its limitations. Although the proposed method is tested with lettuce, it can be potentially applied to other vegetables such as broccoli or sugar beat.

The rest of the paper is organized as follows. In Section 2, related work on crop detection in agriculture and MOT methods are discussed. In Section 3, the experimental setup and the details of the proposed method are described. In Section 4, experimental results of the proposed method and performance comparison with four state-of-the-art MOT methods are presented. In Section 5, conclusions and a discussion about further work are presented.

## 2. Related work

The key aspect for agricultural robots to execute precision spray task is to accurately detect and track each individual plant. Therefore, there are two fields of research that are closely related to our method, which is namely computer vision based crop detection and multiple object tracking.

### 2.1. Crop detection

Crop detection based on computer vision is a key component of precision spray and intelligent weeding systems for agricultural robots. There exist many works of detecting crops using hand-crafted features (Haug et al., 2014; Lottes et al., 2017; Milioto et al., 2017). However, hand-crafted features need to be adjusted differently according to different applications and situations. The disadvantages of using them are being easily affected by illumination and poor robustness. Most traditional methods aim to solve the limitation of information extracted by hand-crafted feature by using complex linear classifiers, e.g., SVM (Guerrero et al., 2012).

In recent years, the progress of the Deep Neural Network (DNN) has led to fundamental changes in all aspects of life. With the development of DNN, the perception capabilities of agricultural robots have been improved significantly (Saleem et al., 2021). Recently, more and more crop weed discrimination and classification methods based on Convolutional Neural Network (CNN) have been proposed and achieved surprising results (Milioto et al., 2018; Su et al., 2021; Ulloa et al., 2022). More abstract and representative information can be extracted through dozens or even hundreds of convolution layers with pooling layers. Jiang et al. (2020) presented GCN-ResNet-101, which is a semi-supervised learning method based on Graph Convolutional Network (GCN), to detect crops and weeds. Recognition accuracies are 97.80, 99.37, 98.93, and 96.51% on four different datasets which include crop and weed with the proposed approach. Ulloa et al. (2022) proposed Convolutional Neural Network (CNN) to detect vegetables and extract geometric characteristics of vegetables, which helped to conduct fertilization operation with the robot arm. Jin et al. (2022) proposed a method of crop-weed detection based on deep learning which can recognize vegetable crops and classify bother green objects as weed. Magalhães et al. (2021) provided an annotated visual dataset containing green and red tomatoes and tested it with five deep learning models. The results show that the single-shot multibox detector can be used to accurately identify targets in the dataset, which helps the harvesting robot to detect tomatoes in real time and *in situ*. Moreira et al. (2022) proposed to utilize a deep learning model to detect tomatoes and classify them to determine their mature stages. The results show that the YOLO-V4 model achieves the best performance with a macro F1-score of 85.81 and 74.16% in the detection and classification tasks, respectively.

In terms of segmentation of vegetable crops, Su et al. (2021) proposed a semantic segmentation algorithm based on DNN to solve the problem of similar appearance between wheat and ryegrass. The algorithm has high segmentation accuracy and can achieve the real-time segmentation performance of 48.95 Frames Per Second (FPS) on Nvidia GTX 1080 GPU to ensure that it can be deployed in real-time. Milioto et al. (2018) proposed a semantic segmentation system using the existing vegetation index to solve the problem of separating beets and weeds in crop fields. This method can achieve real-time classification at the running speed of 20 Hz on a real agricultural robot. You et al. (2020) presented a DNN-based semantic segmentation model, which introduces an attention mechanism to capture long-range contextual information to improve segmentation accuracy. Khan et al. (2020) presented CED-Net, a semantic segmentation approach, that exploits a cascaded encoder-decoder network structure to discriminate between crop and weed.

These methods based on object detection or segmentation can accurately detect and localize all crops in given images. However, they do not solve the correspondence of crops between consecutive images. As a result, conventional robotic precision spray usually requires the robot to travel at a fixed distance so that consecutive images just follow each other without any overlapping or missed crop. This is usually hard for a robot with high autonomy since it might stop or reverse back for dynamic obstacle avoidance. To overcome such a limitation, a better option is to adopt MOT and both detect and track each plant. With each detection assigned with a unique plant ID, the robot ensures to spray each plant exactly once.

### 2.2. Multiple object tracking

Multiple Object Tracking [or Multiple Target Tracking (MTT)] is a very important task in computer vision. Its essence is to detect and locate multiple targets in an image, give them their identities, and maintain their identities in consecutive frames (Luo et al., 2021). At present, advanced online MOT methods can be divided into two categories: two-stages MOT systems (Bewley et al., 2016; Bochinski et al., 2017; Wojke et al., 2017) and one-shot MOT systems (Wu et al., 2021; Zhang et al., 2021a,b; Liang et al., 2022).

The two-stage methods that follow the tracking-by-detection paradigm divide MOT systems into two independent tasks. Detection is first produced by various detector networks, then candidate boxes are added to tracklets across different frames by the data association network. SORT is a simple and fast tracker presented by Bewley et al. (2016) that uses the Kalman filter (Kalman, 1960) to predict the position of the target in the next frame and match it with the detected target with the Hungarian algorithm (Kuhn, 2010). It mainly uses Intersection Over Union (IOU) cost of the predicted bounding box and that of target detection as the basis for data association. However, objects are easy to lose or switch assigned IDs when situations like crowded targets or occlusion between objects happen. In order to solve these problems, DeepSort is proposed by Wojke et al. (2017), which applies a CNN trained with a large-scale person re-identification dataset to extract the appearance information of objects. DeepSort obtains appearance descriptors through a feature embedding to improve the performance of SORT. On the basis of inheriting the motion information of SORT, it combines the motion and appearance information to perform data association. The method is validated to be more effective in solving the problems of object loss, occlusion, and identity switch in complex scenarios. Zhang et al. (2021a) propose ByteTrack, which performs a simple and efficient data association method called BYTE without appearance. In this method, detection boxes with high confidence and low confidence are processed separately, so that the objects in the low score detection boxes are also exploited as much as possible rather than ignored.

Two-stage MOT methods are normally inefficient and slow because the task needs to be processed separately. One-shot MOT methods are introduced to tackle such a limitation. It performs object detection and re-identification (re-ID) feature embedding in separate networks simultaneously. Wang et al. (2020) proposed the first near real-time MOT system, which integrates object detection and appearance feature embedding into one task network. The inference speed of this method can reach from 18.8 FPS to 24.1 FPS when different input resolutions are set. Zhang et al. (2021b) proposed FairMOT, a simple approach that utilizes two homogeneous branches to predict objects and extract re-identification features. Since the unfairness of the two tasks is overcome by this method, it achieves high detection and tracking accuracy on several public MOT datasets. It also verifies that an anchor-free detector is more suitable for identity embedding extraction than an anchor-based detector. The above methods combine detection and feature extraction as one task, but the subsequent data association and matching are still separate tasks. CenterTrack (Zhou et al., 2020) combines detection and tracking into one network and forms an integrated MOT system. It is based on CenterNet (Zhou et al., 2019) which regards the detected objects as points from the detector. The method learns the offset vector between the object center points of two consecutive frames. Greedy matching is performed based on the distance between the predicted offset and the obtained center point in the previous frame for data association. TraDeS (Wu et al., 2021) utilizes tracking clues to assist detection based on CenterTrack (Zhou et al., 2020). It introduces a cost volume-based association module and motion-guided feature warper module to improve tracking accuracy in complex scenarios.

The existing MOT methods extract the feature information of targets to identify the targets that have appeared before. However, these methods tend to fail when the targets disappear in multiple frames or highly similar targets are presented. Unfortunately, these situations are quite common in the case of robotic crop detection and tracking. When the robot needs to reverse back, it observes crops that have been previously observed and lost tracking. Individual crops are also similar in shape, color, and texture. To tackle such challenging scenarios, we propose LettuceTrack, a novel MOT method that exploits the relationship of a plant with its neighbors to improve the accuracy of lettuce detection and tracking for robotic precision spray.

## 3. Materials and methods

When the robot travels along the farm, there exists a relative motion between the camera and the ground, and we adopt vision based detection and tracking to follow each plant. However, the positions of crops are actually immobile relative to the ground. We exploit such a characteristic to build a novel feature for each plant. Together with the proposed matching method, a unique ID for each plant can be reliably established. In the following part of the section, details of the data acquisition, the proposed feature extraction, and data association strategies are illustrated.

### 3.1. Data acquisition

**Figure 2:**
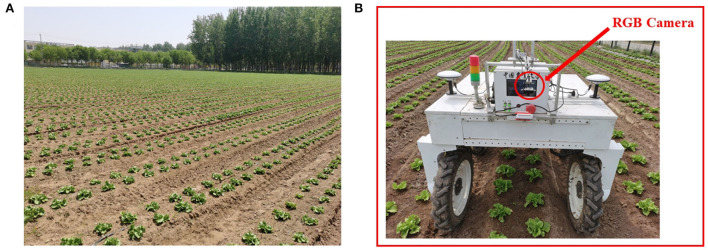
Data acquisition. **(A)** The lettuce farm. **(B)** The agricultural robot capturing images through a downward facing RGB camera.

The data was collected by the authors at a farm in Tongzhou District, Beijing, China. As shown in Figure 2, we used our agricultural robot which is equipped with an RGB camera to capture images when moving in many rows of the farm with lettuce growing in different stages. The speed of the robot varies in different parts of the dataset, which ranges from 0.35 to 0.45 m/s through the entire data acquisition process, according to the feedback data from wheel encoders.

We set the camera angle to be vertically down and at a height of 1.5 m from the ground to ensure that the number of plants in a single column of collected data is greater than three to construct the proposed feature for each plant. This is due to the fact that the proposed feature extraction of a plant is determined by its neighboring plants. The camera is set with a resolution of 1, 920 × 1, 080 and a frequency of 30 Hz. We collected data at two different growth stages of lettuce, which are namely the rosette stage and the heading stage, respectively. Lettuces are in the third and fourth weeks after transplanting. The distance between adjacent plants is from 0.3 to 0.35 m, and the distance between two rows of plants is about 0.3 m. Due to frequent weeding operations, there are fewer weeds, and the maximum weed density is about 10 weeds/m^2^.

**Figure 3:**
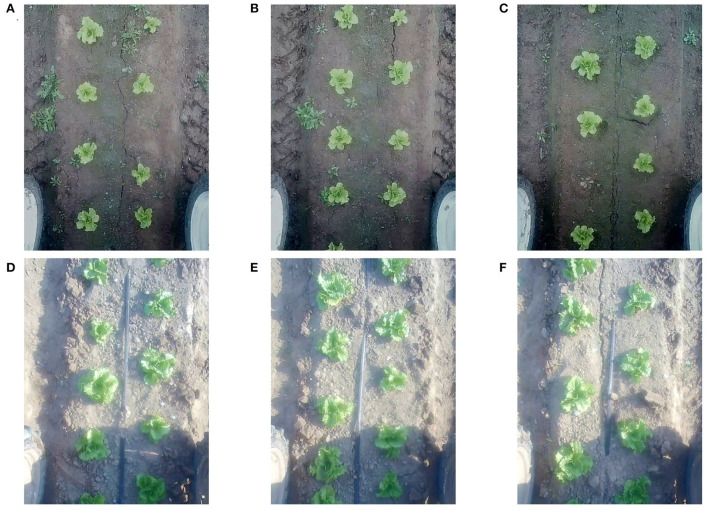
Data acquisition during two growth stages of lettuce. **(A–C)** are lettuces in the third week after transplanting, **(D–F)** are lettuces in the fourth week after transplanting.

There is an obvious difference between plant images at the two growth stages as shown in Figure 3 since the weather and lighting conditions are different at the time of collection. This helps to verify the generality of our method for crops in different growth stages and lighting conditions. The data of each growth stage is divided into one training set and two test sets. The training set is the images collected by the robot traveling straight from the starting point to the end point. The first test set is collected in the same way as the training set. We define this test set as *test*−*straight*. The second test set is collected when the robot travels straight to the end point and then reverses back to the starting point. We define it as *test*- back and forth (B&F). Our method and other state-of-the-art methods are trained and tested on the data of each growth period separately. Left and right parts of images are cropped from the raw camera images to get rid of unrelated area, so the resolution of images decreased from 1, 920 × 1, 080 to 810 × 1, 080. Following the MOT16 (Milan et al., 2016; Dendorfer et al., 2021) dataset, we annotate the six parts of our dataset and obtain ground truth MOT labels, which include the frame, ID number, and bounding box information of every plant. Details about the dataset are summarized in Table 1.

**Table 1:** Summary of six parts of the dataset used in the paper.

**Dataset**	**The rosette stage**			**The heading stage**		
	**Train1**	**Test-straight1^*a*^**	**Test-B&F1^*b*^**	**Train2**	**Test-straight2^*a*^**	**Test-B&F2^*b*^**
Resolution	810 × 1,080	810 × 1,080	810 × 1,080	810 × 1,080	810 × 1,080	810 × 1,080
Length(Frame)	880	545	791	598	873	855
Tracks	191	108	95	106	143	142
Boxes	7,832	4,699	6,707	6,196	8,177	8,021
Application	Train	Test	Test	Train	Test	test

^*a*^The test set *test*−*straight* is the images collected by the robot traveling straight from the starting point to the end point.

^*b*^The test set *test*−*B&F* is collected when the robot travels straight to the end point and then reverses back to the starting point.

### 3.2. Feature extraction and matching

#### 3.2.1. Feature extraction

**Figure 4:**
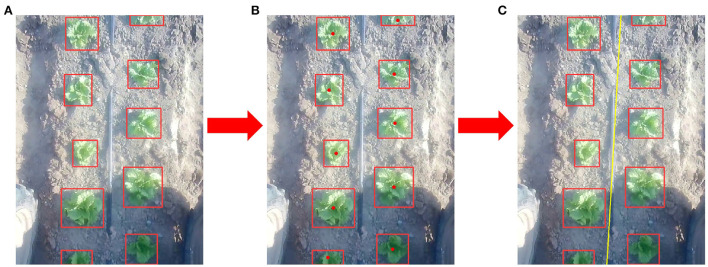
Plants detection and center line extraction. **(A)** Vegetable plants detections. **(B)** Center points extraction. **(C)** Center line (Yellow) extraction.

In the proposed method, a state-of-the-art and light weighted detection method, YOLO-V5, is adopted to detect lettuces (Jubayer et al., 2021; Zhao et al., 2021; Wang et al., 2022). Then, we can get the bounding box of each object in one frame and calculate the center point of each bounding box. As shown in Figure 4, a center line can be fitted through center points of detected plants as follows,


(1) $$\begin{array}{l} x=k\cdot y+b. \end{array}$$

Once the center line is determined, plants can be divided into different lanes. Suppose there are two lanes on the farm, then two plants, whose center points of bounding boxes are (*x*_1_, *y*_1_) and (*x*_2_, *y*_2_), respectively, are in the same lane if their center points satisfy,


(2) $$\begin{array}{l} \left(k\cdot y_{1}+b-x_{1}\right)\cdot \left(k\cdot y_{2}+b-x_{2}\right)>0. \end{array}$$

If there are multiple lanes, plants at each lane can be determined judging from their distance to the center line.

**Figure 5:**
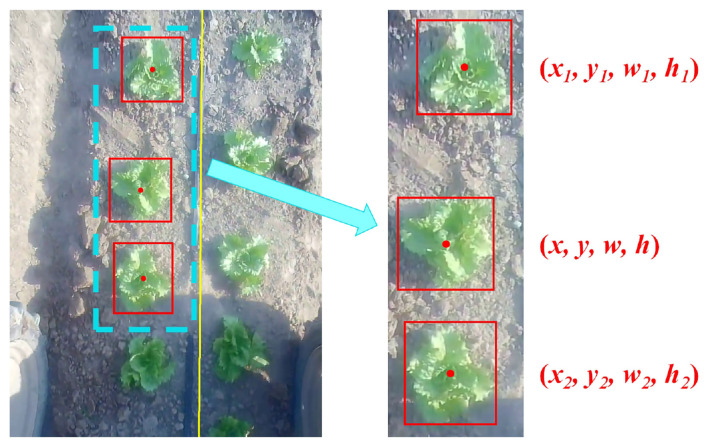
Feature generation for a plant. To construct the feature for the second plant from the top on the left line, detection results of the plant above it, the plant below it, and itself are utilized. The feature is specifically defined by Equation (3).

To identify each individual plant, a novel geometric feature is generated for the plant. As all plants are fixed on the farm, we exploit this characteristic and design the feature based on its relationship with its neighboring plants at the same line. Take the second plant from the top on the left line in Figure 5 as an example, its feature is determined by the plant above it, the plant below it, and itself. We will concentrate on these three plants to illustrate the generation of plant features. From the detection results, we can obtain the coordinates of the center point, width, and height of each bounding box. The coordinates, widths, and heights of the middle plant, the upper plant, and the lower plant are expressed as (*x, y, w, h*), (*x*_1_, *y*_1_, *w*_1_, *h*_1_), and (*x*_2_, *y*_2_, *w*_2_, *h*_2_), respectively. Finally, the feature of each plant *F* can be constructed as follows:


(3) $$\begin{array}{l} F=\left[\begin{array}{c} d_{1}\\ d_{2}\\ w_{r}\\ h_{r} \end{array}\right]=\left[\begin{array}{c} c_{1}\cdot \sqrt{{\left(x_{1}-x\right)}^{2}+{\left(y_{1}-y\right)}^{2}}\\ c_{2}\cdot \sqrt{{\left(x_{2}-x\right)}^{2}+{\left(y_{2}-y\right)}^{2}}\\ c_{w}\cdot \frac{w_{1}}{w_{2}}\\ c_{h}\cdot \frac{h_{1}}{h_{2}} \end{array}\right], \end{array}$$

where *d*_1_ and *d*_2_ are the distances from the center point of the upper and lower bounding boxes to the center point of the middle bounding box, respectively. *w*_*r*_ and *h*_*r*_ are the width ratio and height ratio between the upper and lower bounding boxes. In order to balance the influences of different parts of the feature vector, we multiply them with weighting parameters *c*_1_, *c*_2_, *c*_*w*_, and *c*_*h*_. These parameters control the importance of two distances and two ratios during the feature matching later. *c*_*w*_ and *c*_*h*_ can be tuned slightly larger to balance the influence of the distance and the ratio.

#### 3.2.2. Data association

Once the feature for each plant is computed as described in the previous section, it can be used to match plants in the current image to those in the previous image. Specifically, the distance between two features $F^{1}$ and $F^{2}$ are defined as follows:


(4) $$\begin{array}{l} dist(F^{1},F^{2})=\sqrt{{\left(F^{1}-F^{2}\right)}^{T}(F^{1}-F^{2})}\\ \;=\sqrt{\begin{array}{l} {\left(d_{1}^{1}-d_{1}^{2}\right)}^{2}+{\left(d_{2}^{1}-d_{2}^{2}\right)}^{2}+{\left(w_{r}^{1}-w_{r}^{2}\right)}^{2}\\ +{\left(h_{r}^{1}-h_{r}^{2}\right)}^{2} \end{array}}, \end{array}$$

where $F^{1}$ and $F^{2}$ are feature vectors of two detected plants as defined in Equation (3), and $d_{1}^{1}$, $d_{2}^{1}$, $w_{r}^{1}$, $h_{r}^{1}$, and $d_{1}^{2}$, $d_{2}^{2}$, $w_{r}^{2}$, and $h_{r}^{2}$ are corresponding feature elements. In essence, Euclidean distance is used to evaluate feature similarity of two detected plants for data association. If two targets involved in the comparison are the same target, the calculated distance in Equation (4) is less than a predefined threshold. Based on the feature distance, we construct a feature cost matrix denoted as **Matrix_feat_** to perform the association of the targets in the later stage.

In addition to feature distance, we also utilize the Kalman filter (Kalman, 1960) to predict the positions of plants in the current frame according to those in the previous frame. We calculate the IOU of the predicted bounding box from the Kalman filter and the bounding box from the detection result to construct an IOU cost matrix denoted as **Matrix_IOU_**. We perform subtraction operation on two matrices as follows to get the final cost matrix denoted as **Matrix_final_**,


(5) $$Matrix_{final}=Matrix_{feat}-Matrix_{IOU}.$$

When two plants have a smaller feature distance and larger IOU, the cost matrix **Matrix_final_** has a smaller value at the corresponding element, which means those two plants are more likely to be one plant. **Matrix_final_** has better matching accuracy than using **Matrix_feat_** and **Matrix_IOU_** alone. Finally, Hungarian algorithm (Kuhn, 2010) is deployed for an association of various plants based on the **Matrix_final_**.

**Figure 6:**
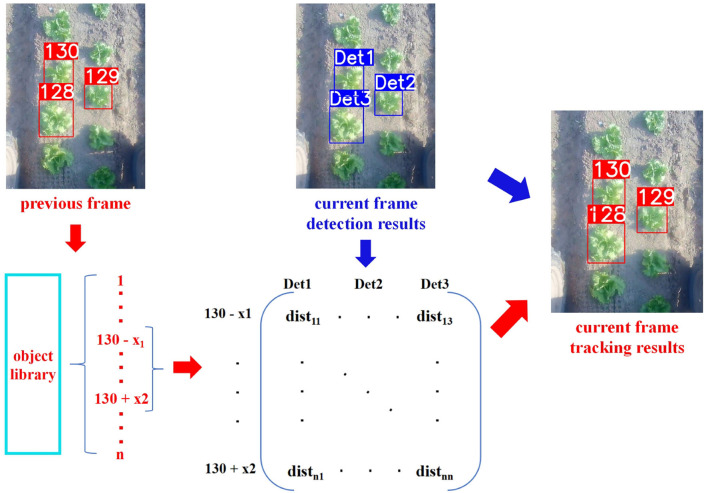
The structure of the proposed data association method based on the proposed feature extraction.

In order to tackle the situation of re-identifying a plant that goes out of the camera field of view for a long time and re-appears in the current frame, an object library is built to store the plants that have appeared before. The plants in the object library are ordered by their ID numbers. When constructing the cost matrix **Matrix_feat_**, **Matrix_IOU_**, and **Matrix_final_**, match candidates are searched from neighbors around the biggest ID that appeared in the previous frame. If the matching cost is larger than a predefined threshold, a new ID is assigned. An example is shown in Figure 6, there are three plants in the middle part of images of the previous frame and the current frame whose plant feature can be extracted as stated in Section 3.2.1. Since the biggest ID in the previous frame is 130, when constructing the cost matrix, matching candidates are searched from the neighbors of 130, i.e., from 130−*x*_1_ to 130+*x*_2_. Then, the cost matrix **Matrix_final_** is computed between the detected plants with the proposed feature, i.e., Det1, Det2, and Det3, and plants from 130−*x*_1_ to 130+*x*_2_ in the object library according to Equation (5). After applying the Hungarian method, Det1, Det2, and Det3 are matched to ID 130, 129, and 128, respectively.

Finally, we focus on the plants on top and bottom of the images, whose features cannot be extracted as described in Section 3.2.1, since they do not have complete top or bottom neighbors. To assign IDs to these plants, first, the travel direction of the robot is determined by comparing image coordinates of plants in the middle part of images that have been successfully detected and tracked. Then, those plants which are going to go out of the camera's field of view are matched with plants in the previous frame. Those plants which are newly appeared in the camera field of view are further divided into new cases. If the ID of the nearest successfully detected and tracked plant in the middle part of the image is equal to the maximum ID of the object library, then a new ID is assigned to the newly appeared plant. Otherwise, they are matched with local neighbors of plants in the middle.

**Figure 7:**
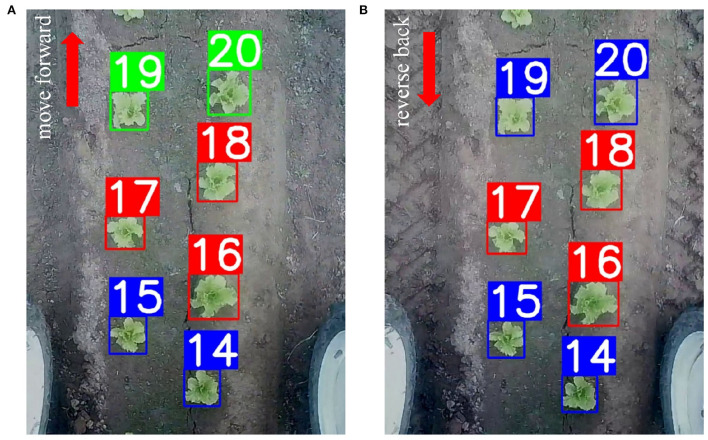
ID assignment for plants whose features cannot be extracted as described in Section 3.2.1. In **(A)**, the robot travels forward from the starting point until the plant 20, and in **(B)**, it keeps moving forward until the plant 30 and moves backward to the plant 20. Red rectangles represent plants whose features can be extracted as described in Section 3.2.1, blue rectangles denote plants that are matched with previously appeared plants, and green rectangles denote plants that are assigned with new IDs.

Two examples are shown in Figure 7. The robot travels forward from the starting point until the plant with ID 20 in Figure 7A, then it keeps traveling until the plant with ID 30, and then reverses back to the plant with ID 20 in Figure 7B. In both images, red rectangles denote plants whose features can be extracted as described in Section 3.2.1, blue rectangles denote plants that are matched with previously appeared plants, and green rectangles denote plants that are assigned with new IDs. In Figure 7A, the robot travels up, so plants with IDs 14 and 15 are matched with plants in the previous frame. Similarly in Figure 7B, the robot travels down, then plants with IDs 19 and 20 can also be matched with plants in the previous frame in the same way. Regarding plants that are newly appeared in Figure 7A, since the object library has the maximum ID of 18, which is equal to the ID of plant 18 in the current frame, new IDs of 19 and 20 are assigned to these plants. However, in Figure 7B, the maximum ID of the object library is 30 which is different from the ID of plant 16 in the current frame, they are matched with neighbors of plant 16, and then matched to plants with IDs of 14 and 15.

## 4. Experimental results

In this section, implementation details of the proposed method, evaluation metrics for MOT accuracy, results of the proposed method, and its comparison with four state-of-the-art methods, as well as limitations of the proposed method are discussed.

### 4.1. Implementation details

As mentioned before, YOLO-V5 is employed as the detector in our method. Specifically, we choose to use the YOLO-V5m model of YOLO-V5 as our detector because it has both high inference speed and detection accuracy. It is trained on two parts of training data corresponding to two growth stages of lettuces in Table 1 based on the pre-trained model on COCO dataset with the SGD optimizer for 150 epochs. A NVIDIA RTX 2080Ti GPU is used for training and inference. The learning rate is initialized with 1*e*^−2^, and the input resolution of the neural net is set to be 640 × 640.

For the other four state-of-the-art MOT methods, which are ByteTrack, FairMOT, TraDeS, and SORT^1^, we finetune them on our dataset using their default hyperparameters. We conduct 150 epochs of training for each method on the pretrained model provided by the authors.

### 4.2. Evaluation metrics

The evaluation of MOT task is more complex than the detection and segmentation task. Multiple Object Tracking Accuracy (MOTA) (Bernardin and Stiefelhagen, 2008) is commonly used in many existing MOT works, but it is also shown to be affected by the detection and cannot well reflect the quality of data association in a method. To resolve this, Ristani et al. (2016) proposed identity related measures, i.e., Identification Recall (IDR), Identification Precision (IDP), and IDF1, which can better reflect the performance of data association. Formulations of IDR, IDP, and IDF1 are summarized as follows,


(6) $$\begin{array}{l} IDF1=\frac{2IDTP}{2IDTP+IDFP+IDFN}, \end{array}$$

(7) $$\begin{array}{l} IDR=\frac{IDTP}{IDTP+IDFN}, \end{array}$$

(8) $$\begin{array}{l} IDP=\frac{IDTP}{IDTP+IDFP}, \end{array}$$

where IDTP, IDFN, and IDFP refer to the number of true positive, false negative, and false positive ID assignment, respectively. In addition, ID Switch (IDSW) (Bernardin and Stiefelhagen, 2008; Li et al., 2009) is proposed to measure the stability of tracking.

Another popular metrics for evaluating MOT accuracy is Higher Order Tracking Accuracy (HOTA) presented by Luiten et al. (2021), which balances between detection and association performance. HOTA is calculated by detection accuracy score (DetA) and association accuracy score (AssA) as follows,


(9) $$\begin{array}{l} HOTA=\sqrt{DetA\cdot AssA}. \end{array}$$

Among them, AssA is a combination of association accuracy score (AssRe) and association precision (AssPr) as follows,


(10) $$\begin{array}{l} AssA=\frac{AssRe\cdot AssPr}{AssRe+AssPr-AssRe\cdot AssPr}, \end{array}$$

where AssRe reflects the proportion of predicted trajectories in ground truth trajectories, and AssPr measures the accuracy of predicted trajectories tracking the trajectories in the ground truth. The detailed description of DetA, AssA, AssRe, and AssPr can be found in the original work (Luiten et al., 2021), which is omitted here for the brevity of the paper. In general, HOTA can better reflect the human's visual perception for MOT evaluation.

In this paper, we compute the above-mentioned MOT evaluation metrics with the MOTChallenge official kit^2^ (Dendorfer et al., 2021).

### 4.3. Results and discussions

We evaluate the MOT performance of the proposed method and four state-of-the-art methods with our dataset using the evaluation metrics mentioned above. The results are summarized in Table 2. In the table, *test*−*straight*1 and *test*−*straight*2, *test*−*B&F*1, and *test*−*B&F*2 indicate the situations where the robot travels only forward and the situations where the robot travels both forward and backward in the first and second growth stages, respectively.

**Table 2:** Performance of the proposed method and comparison to four state-of-the-art Multiple Object Tracking (MOT) methods.

**Dataset**	**Method**	**IDSW^a^ ↓**	**HOTA(%) ↑**	**DetA(%) ↑**	**AssA(%) ↑**	**AssRe(%) ↑**	**AssPr(%) ↑**	**IDF1(%) ↑**	**IDR(%) ↑**	**IDP(%) ↑**	**FPS ↑**
Test-straight1	ByteTrack	**0**	61.010	58.492	64.246	67.990	86.835	83.586	72.824	**98.080**	30.13
	FairMOT	57	75.800	76.029	75.889	78.878	89.468	91.261	87.338	95.553	27.04
	TraDeS	59	69.292	**82.572**	58.675	80.109	67.936	71.471	69.121	73.986	23.84
	Sort	**0**	**80.014**	79.661	**80.337**	**84.330**	91.313	**94.011**	**90.360**	97.970	**98.281**
	ours	10	77.589	79.405	75.814	76.860	**95.734**	86.053	77.336	96.984	91.85
Test-B&F1	ByteTrack	89	45.963	58.947	36.439	37.775	85.711	50.661	44.849	58.204	29.69
	FairMOT	1,710	40.203	63.200	25.878	26.571	75.102	41.858	36.753	48.610	28.57
	TraDeS	220	47.119	**83.570**	26.866	42.121	58.283	45.782	44.670	46.952	23.21
	Sort	86	58.314	78.574	43.301	44.386	91.722	54.283	51.633	57.221	**98.355**
	ours	**53**	**76.809**	79.691	**74.032**	**75.423**	**94.496**	**85.295**	**76.756**	**95.973**	91.62
Test-straight2	ByteTrack	**0**	55.553	53.609	57.860	62.545	83.010	76.976	66.687	91.020	29.63
	FairMOT	17	71.706	70.980	72.810	75.893	88.247	90.674	84.958	97.215	29.09
	TraDeS	10	63.437	**88.991**	45.361	**90.234**	48.031	51.250	50.642	51.873	24.57
	Sort	**0**	**78.080**	77.733	**78.462**	82.992	88.878	**94.116**	**90.962**	97.496	**97.717**
	ours	3	71.617	71.255	71.987	72.319	**98.974**	84.070	72.545	**99.949**	92.00
Test-B&F2	ByteTrack	131	37.297	46.163	31.227	32.528	82.975	45.124	37.639	56.325	29.51
	FairMOT	1,102	42.513	63.676	28.730	29.586	81.424	41.663	37.240	47.278	27.65
	TraDeS	187	52.445	**89.490**	30.823	45.931	58.509	50.359	49.882	50.845	23.36
	Sort	133	52.812	71.886	38.871	39.981	88.304	48.362	45.194	52.009	**97.108**
	ours	**50**	**70.315**	72.238	**68.445**	**69.527**	**95.551**	**81.857**	**70.876**	**96.865**	89.80

^*a*^Symbols ↑ and ↓ after the evaluation metrics indicate the value of it is the higher the better or the lower the better, respectively. The bold numbers show the best performing method.

It can be seen from the table that SORT performs the best among other methods overall in terms of HOTA and IDSW, in the test data *test*−*straight*1 and *test*−*straight*2 where the robot only moves forward. Our method is slightly worse than SORT but better or similar to other methods. It is because this is a simple situation where all plants move in one direction in captured images, and SORT is especially suitable for such cases. Other state-of-the-art methods like FairMOT and TraDeS try to extract plant features for re-identification. However, different from human tracking, individual plants are visually quite similar to each other in terms of both color and texture. Therefore, the advanced object feature extraction and matching for object re-identification parts of FairMOT and TraDeS sometime provide misleading information. Our method also performs feature extraction and matching, but our feature extraction is based on the geometric relationship of a plant with its neighbors. Therefore, it provides better differentiation than the image feature of an individual plant, thereby suffering less from similar appearance of plants.

In the test data *test*−*B&F*1 and *test*−*B&F*2 where the robot moves both forward and backward, our method shows significantly better performance than other state-of-the-art methods, thanks to the proposed feature extraction and data association strategies. Other state-of-the-art methods cannot handle the situation where a plant disappeared from the camera field of view a long time ago and re-appears again and will assign new a ID to this plant. However, the proposed method can successfully search and re-identify the plant from its object library by comparing the proposed feature. For the robotic precision spray application, this is quite meaningful since assigning a new ID to the same plant means spraying the same plant twice.

**Figure 8:**
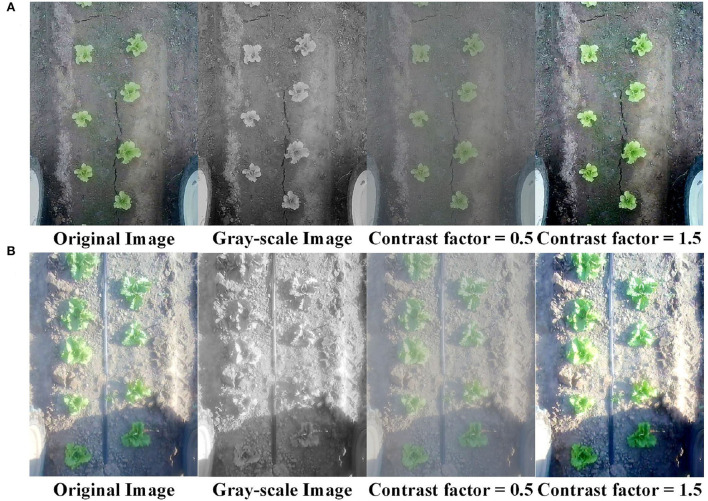
Images in the dataset with different color contrasts. **(A,B)** are images of lettuces in the rosette stage and the heading stage. Images from left to right correspond to the original images, the gray-scale images, images with a contrast factor of 0.5, and images with a contrast factor of 1.5, respectively.

In addition, to investigate the impact of the color contrast of the captured images on the performance of the proposed method, experiments are conducted by changing the color contracts of all images in the dataset. As shown in Figure 8, we change the original images in the dataset to be grayscale images, images with a contrast factor of 0.5 and images with a contrast factor of 1.5. The proposed method is trained and tested on the dataset with different color contrasts independently, and the results are summarized in Table 3. We can see from the table that in the test data of *test*−*straight*1 and *test*−*B&F*1, the performances of our method with images of different color contrasts are quite similar. In test data of *test*−*straight*2 and *test*−*B&F*2, the performance of our method with the gray-scale images is noticeably lower than those of the other three. This is mainly because there exists a certain level of over exposure in the captured images of *test*−*straight*2 and *test*−*B&F*2, which increase the difficulty of detection, especially with the grayscale images, as shown in Figure 8B. In comparison, lettuces are more clear in the grayscale images of *test*−*straight*1 and *test*−*B&F*1, as shown in Figure 8A. In summary, the performance of the proposed method is generally similar with images of different color contrasts, when captured images are clear and not overexposed. However, when the images of lettuces are not very clear, e.g., *when they are overexposed, the performance tends to degrade especially with the grayscale images*.

**Table 3:** Performance of the proposed method with images of different color contrasts.

**Dataset**	**Image Tpye**	**IDSW^a^ ↓**	**HOTA(%) ↑**	**DetA(%) ↑**	**AssA(%) ↑**	**AssRe(%) ↑**	**AssPr(%) ↑**	**IDF1(%) ↑**	**IDR(%) ↑**	**IDP(%) ↑**	**FPS ↑**
Test-straight1	Gray-scale Image	**10**	77.530	79.376	75.728	76.809	95.691	**86.066**	**77.357**	**96.985**	91.09
	Image with contrast factor of 0.5	22	76.488	79.408	73.677	75.409	93.258	84.409	75.867	95.117	91.22
	Image with contrast factor of 1.5	13	**77.592**	**79.453**	75.775	76.814	95.698	85.995	77.293	96.905	90.47
	Original Image	**10**	77.589	79.405	**75.814**	**76.860**	**95.734**	86.053	77.336	96.984	**91.85**
Test-B&F1	Gray-scale Image	**36**	**77.922**	79.798	**76.090**	77.245	**95.679**	**86.692**	**78.038**	**97.504**	90.65
	Image with contrast factor of 0.5	46	77.511	79.771	75.316	76.495	95.354	86.121	77.531	96.852	**93.07**
	Image with contrast factor of 1.5	46	77.526	**79.863**	75.256	76.613	94.854	86.166	77.591	96.873	90.09
	Original Image	53	76.809	79.691	74.032	75.423	94.496	85.295	76.756	95.973	91.62
Test-straight2	Gray-scale Image	19	66.250	66.352	66.303	67.717	92.335	82.240	71.126	97.469	91.92
	Image with contrast factor of 0.5	**3**	**71.710**	**71.367**	**72.060**	**72.422**	98.883	**84.202**	**72.741**	**99.950**	88.51
	Image with contrast factor of 1.5	**3**	71.083	70.730	71.450	71.889	98.468	83.987	72.447	99.899	90.32
	Original Image	**3**	71.617	71.255	71.987	72.319	**98.974**	84.070	72.545	99.949	**92.00**
Test-B&F2	Gray-scale Image	197	62.834	67.143	58.908	61.252	86.497	77.363	67.149	91.242	87.74
	Image with contrast factor of 0.5	**45**	**70.688**	**72.256**	**69.156**	**69.901**	**96.457**	**82.190**	**71.201**	**97.192**	86.79
	Image with contrast factor of 1.5	62	69.719	71.904	67.604	68.788	94.844	81.475	70.540	96.421	84.77
	Original Image	50	70.315	72.238	68.445	69.527	95.551	81.857	70.876	96.865	**89.80**

^*a*^Symbols ↑ and ↓ after the evaluation metrics indicate the value of it is the higher the better or the lower the better, respectively. The bold numbers show the best performing method.

**Figure 9:**
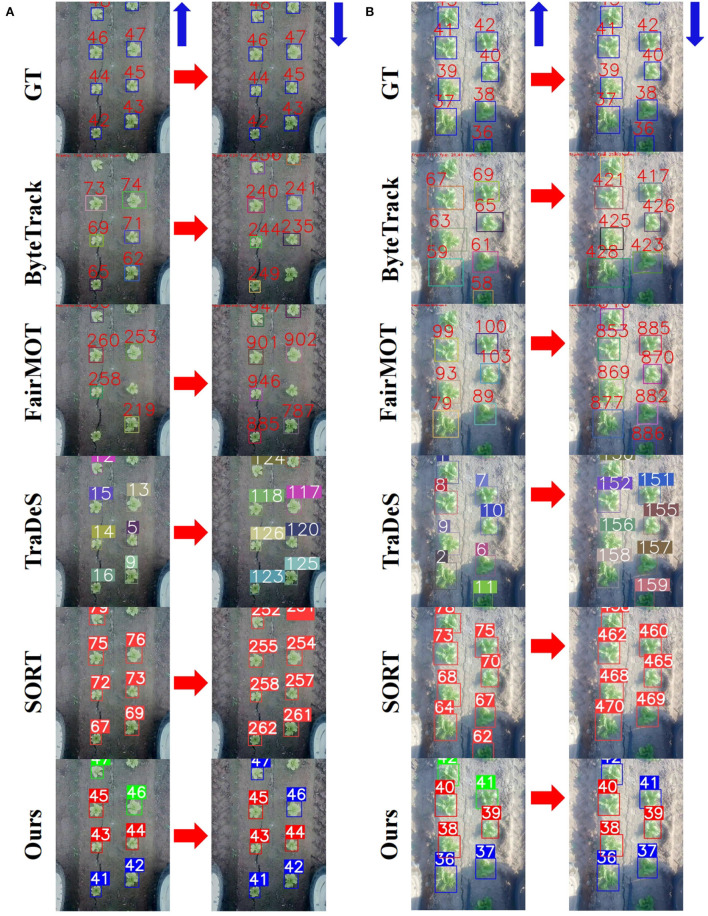
Qualitative comparisons of the proposed method and other state-of-the-art methods in the test data *test*−*B&F*1 and *test*−*B&F*2 where the robot moves both forward and backward. **(A)** results on *test*−*B&F*1 and **(B)** results *test*−*B&F*2. The left and right columns of **(A,B)** show images of the same plants when the robot moves forward and reverses back.

Qualitative comparisons of MOT performance between the proposed method and other state-of-the-art methods in the test data *test*−*B&F*1 and *test*−*B&F*2 are shown in Figure 9. In both Figures 9A,B, the blue arrows in the figure indicate the direction of robot motion. Specifically, the robot moves forward to a place and captures images in the left columns. It continues its travel for a while, then reverses back, comes to the same place, and captures images in the right columns of Figures 9A,B. Thus, the left and right columns show images of the same plants when the robot moves forward and reverses back. It can be seen that only the proposed method successfully re-identifies the same plants, while other methods assign new IDs for them. Note that in the left columns of Figures 9A,B, although SORT, as well as other methods, shows different ID numbers to ground truth ID labels, it does not necessarily mean the assigned ID is incorrect. In fact, as long as IDs for plants are consistent during the whole process, the result is acceptable.

The inference speed is shown in Table 2 in terms of inference FPS. We can see from the table that the FPS of SORT is the highest among others since it does not need to extract object features. Although our method also extracts features of plants and perform data association, this process takes very little time, and it is only less than 10% slower than SORT while significantly better than other methods. Since the average FPS of the proposed method is approximately 90 FPS, it well meets the requirements of the real-time robotic spray action.

### 4.4. Limitations

There are two limitations that exist in the proposed methods. First, our method assumes that the positions of targets to be detected and tracked are fixed on the ground. While it is obviously true for robotic precision spray application, it is not the general case of MOT in computer vision society, but a special case of it. Second, it can be seen from the experimental results that the performance of the proposed method is similar to or a little worse than the best performing method, SORT, when the robot travels forward only. Its advantages over other state-of-the-art methods become obvious when the robot moves back and forth, which is quite normal in reality, e.g., it needs to avoid dynamic obstacles.

## 5. Conclusions

In this paper, an MOT method, LettuceTrack, for detection and tracking of lettuces is presented to solve robotic precision spray application. We propose a novel feature extraction and data association strategy to re-identify plants which go out of the camera's field of view and re-appear again. This ensures the robot to correctly recognize the same plant and spray them only once when it needs to reverse back for different reasons. Experimental validation of the proposed method is conducted using the dataset collected by our agricultural robot on a lettuce farm, and a comparison with other state-of-the-art methods has been provided. The results show that the proposed method shows superior performance to other methods by successfully re-identifying the same plants when the robot travels back and forth. The proposed method also runs at a high-speed of 90 FPS, which confirms its real-time deployment at the camera frame rate, i.e., around 30 FPS. Furthermore, limitations of the proposed method are also provided. The future work is to find a global re-identification strategy for the robot to recognize the same plants when it completely moves out of the farm and re-enters it again.

## Data availability statement

The raw data supporting the conclusions of this article will be made available by the authors, without undue reservation.

## Author contributions

NH, DS, SW, and PN contributed to the conception and design of the study. NH, SW, and YC organized the experimental dataset. NH performed the statistical analysis. NH and DS wrote the first draft of the manuscript. PN, YQ, and YJ improved the algorithm and wrote sections of the manuscript. All authors contributed to manuscript revision, read, and approved the submitted version.

## Funding

This research was financially supported by the National Natural Science Foundation of China (Grant No. 3217150435) and China Agricultural University with Global Top Agriculture-related Universities International Cooperation Seed Fund 2022.

## Conflict of interest

The authors declare that the research was conducted in the absence of any commercial or financial relationships that could be construed as a potential conflict of interest.

## Publisher's note

All claims expressed in this article are solely those of the authors and do not necessarily represent those of their affiliated organizations, or those of the publisher, the editors and the reviewers. Any product that may be evaluated in this article, or claim that may be made by its manufacturer, is not guaranteed or endorsed by the publisher.

## Footnotes

^1^The following open source implementations are used in the experiment. ByteTrack: https://github.com/ifzhang/ByteTrack, FairMOT: https://github.com/ifzhang/FairMOT, TraDeS: https://github.com/JialianW/TraDeS and SORT: https://github.com/abewley/sort.

^2^ https://github.com/JonathonLuiten/TrackEval

## References

[B1] Adamides G. Katsanos C. Constantinou I. Christou G. Xenos M. Hadzilacos T.. (2017). Design and development of a semi-autonomous agricultural vineyard sprayer: human-robot interaction aspects. J. Field Rob. 34, 1407–1426. 10.1002/rob.21721

[B2] Bac C. W. Hemming J. van Tuijl B. A. J. Barth R. Wais E. van Henten E. J. (2017). Performance evaluation of a harvesting robot for sweet pepper. J. Field Rob. 34, 1123–1139. 10.1002/rob.21709

[B3] Bargoti S. Underwood J. P. (2017). Image segmentation for fruit detection and yield estimation in apple orchards. J. Field Rob. 34, 1039–1060. 10.1002/rob.21699

[B4] Bernardin K. Stiefelhagen R. (2008). Evaluating multiple object tracking performance: the clear mot metrics. EURASIP J. Image Video Process. 2008, 246309. 10.1155/2008/246309

[B5] Bewley A. Ge Z. Ott L. Ramos F. T. Upcroft B. (2016). Simple online and realtime tracking, in 2016 IEEE International Conference on Image Processing (ICIP 2016) (Phoenix, AZ: IEEE), 3464–3468.

[B6] Bochinski E. Eiselein V. Sikora T. (2017). High-speed tracking-by-detection without using image information, in International Workshop on Traffic and Street Surveillance for Safety and Security at IEEE AVSS 2017 (Lecce: IEEE), 1–6.

[B7] Chebrolu N. Lottes P. Schaefer A. Winterhalter W. Burgard W. Stachniss C. (2017). Agricultural robot dataset for plant classification, localization and mapping on sugar beet fields. Int. J. Rob. Res. 36, 1045–1052. 10.1177/0278364917720510

[B8] Dendorfer P. Osep A. Milan A. Schindler K. Cremers D. Reid I. D.. (2021). Motchallenge: a benchmark for single-camera multiple target tracking. Int. J. Comput. Vis. 129, 845–881. 10.1007/s11263-020-01393-0

[B9] Dong J. Burnham J. G. Boots B. Rains G. Dellaert F. (2017). 4D crop monitoring: Spatio-temporal reconstruction for agriculture, in 2017 IEEE International Conference on Robotics and Automation (ICRA 2017) (Singapore: IEEE), 3878–3885.

[B10] Guerrero J. M. Pajares G. Montalvo M. Romeo J. Guijarro M. (2012). Support vector machines for crop/weeds identification in maize fields. Expert. Syst. Appl. 39, 11149–11155. 10.1016/j.eswa.2012.03.040

[B11] Haug S. Michaels A. Biber P. Ostermann J. (2014). Plant classification system for crop/weed discrimination without segmentation, in 2014 IEEE Winter Conference on Applications of Computer Vision (Steamboat Springs, CO: IEEE), 1142–1149.

[B12] Jiang H. Zhang C. Qiao Y. Zhang Z. Zhang W. Song C. (2020). Cnn feature based graph convolutional network for weed and crop recognition in smart farming. Comput. Electron. Agric. 174, 105450. 10.1016/j.compag.2020.105450

[B13] Jin X. Sun Y. Che J. Bagavathiannan M. V. Yu J. Chen Y. (2022). A novel deep learning-based method for detection of weeds in vegetables. Pest Manag. Sci. 78, 1861–1869. 10.1002/ps.6804 35060294

[B14] Jubayer F. Soeb J. A. Mojumder A. N. Paul M. K. Barua P. Kayshar S.. (2021). Detection of mold on the food surface using yolov5. Curr. Res. Food Sci. 4, 724–728. 10.1016/j.crfs.2021.10.003 34712960 PMC8529025

[B15] Kalman R. E. (1960). A new approach to linear filtering and prediction problems. J. Basic Eng. 82D, 35–45. 10.1115/1.366255230253628

[B16] Khan A. Ilyas T. Umraiz M. Mannan Z. I. Kim H. (2020). Ced-net: crops and weeds segmentation for smart farming using a small cascaded encoder-decoder architecture. Electronics 9, 1602. 10.3390/electronics9101602

[B17] Kuhn H. W. (2010). The hungarian method for the assignment problem. Naval Res. Logist. 52, 7–21. 10.1002/nav.20053

[B18] Kurita H. Iida M. Cho W. Suguri M. (2017). Rice autonomous harvesting: operation framework. J. Field Rob. 34, 1084–1099. 10.1002/rob.21705

[B19] Lee J. J. H. Frey K. Fitch R. Sukkarieh S. (2014). Fast path planning for precision weeding, in 2014 Australasian Conference on Robotics and Automation (ACRA 2014) (Melbourne, VIC).

[B20] Li Y. Huang C. Nevatia R. (2009). Learning to associate: hybridboosted multi-target tracker for crowded scene, in 2009 IEEE Conference on Computer Vision and Pattern Recognition(CVPR 2009) (Miami, FL: IEEE), 2953–2960.

[B21] Liang C. Zhang Z. Lu Y. Zhou X. Li B. Ye X.. (2022). Rethinking the competition between detection and reid in multiobject tracking. IEEE Trans. Image Process. 31, 3182–3196. 10.1109/TIP.2022.3165376 35412982

[B22] Lottes P. Khanna R. Pfeifer J. Siegwart R. Stachniss C. (2017). UAV-based crop and weed classification for smart farming, in 2017 IEEE International Conference on Robotics and Automation (ICRA 2017) (Singapore: IEEE), 3024–3031.

[B23] Luiten J. Osep A. Dendorfer P. Torr P. H. S. Geiger A. Leal-Taixé L.. (2021). Hota: a higher order metric for evaluating multi-object tracking. Int. J. Comput. Vis. 129, 548–578. 10.1007/s11263-020-01375-2 33642696 PMC7881978

[B24] Luo W. Xing J. Milan A. (2021). Multiple object tracking: a literature review. Artif. Intll. 293, 103448. 10.1016/j.artint.2020.103448

[B25] Magalh aes S. A. Castro L. Moreira G. dos Santos F. N. Cunha M. Dias J.. (2021). Evaluating the single-shot multibox detector and yolo deep learning models for the detection of tomatoes in a greenhouse. Sensors 21, 3569. 10.3390/s21103569 34065568 PMC8160895

[B26] McCool C. Beattie J. Firn J. Lehnert C. Kulk J. Bawden O.. (2018). Efficacy of mechanical weeding tools: a study into alternative weed management strategies enabled by robotics. IEEE Rob. Autom. Lett. 3, 1184–1190. 10.1109/LRA.2018.2794619

[B27] Milan A. Leal-Taixé L. Reid I. D. Roth S. Schindler K. (2016). Mot16: a benchmark for multi-object tracking. ArXiv, abs/1603.00831. 10.48550/arXiv.1603.00831

[B28] Milioto A. Lottes P. Stachniss C. (2017). Real-time blob-wise sugar beets vs weeds classification for monitoring fields using convolutional neural networks, in 2017 ISPRS Annals of the Photogrammetry, Remote Sensing and Spatial Information Sciences (Wuhan), 41.

[B29] Milioto A. Lottes P. Stachniss C. (2018). Real-time semantic segmentation of crop and weed for precision agriculture robots leveraging background knowledge in CNNs, in 2018 IEEE International Conference on Robotics and Automation (ICRA 2018) (Brisbane, QLD: IEEE), 2229–2235.

[B30] Moreira G. Magalhães S. A. Pinho T. dos Santos F. N. Cunha M. (2022). Benchmark of deep learning and a proposed hsv colour space models for the detection and classification of greenhouse tomato. Agronomy 12, 356. 10.3390/agronomy12020356

[B31] Ristani E. Solera F. Zou R. S. Cucchiara R. Tomasi C. (2016). Performance measures and a data set for multi-target, multi-camera tracking, in 2016 European Conference on Computer Vision(ECCV 2016) (Amsterdam), 17–35.

[B32] Ruckelshausen A. Biber P. Dorna M. Gremmes H. Klose R. Linz A.. (2009). BoniRob: an autonomous field robot platform for individual plant phenotyping. Precision Agric. 9, 841–847.

[B33] Sa I. Lehnert C. English A. McCool C. Dayoub F. Upcroft B.. (2017). Peduncle detection of sweet pepper for autonomous crop harvesting-Combined Color and 3-D Information. IEEE Rob. Autom. Lett. 2, 765–772. 10.1109/LRA.2017.2651952

[B34] Saleem M. H. Potgieter J. Arif K. M. (2021). Automation in agriculture by machine and deep learning techniques: a review of recent developments. Precision Agric. 22, 2053–2091. 10.1007/s11119-021-09806-x

[B35] Su D. Qiao Y. Kong H. Sukkarieh S. (2021). Real time detection of inter-row ryegrass in wheat farms using deep learning. Biosyst. Eng. 204, 198–211. 10.1016/j.biosystemseng.2021.01.019

[B36] Ulloa C. C. Krus A. Barrientos A. del Cerro J. Valero C. (2022). Robotic fertilization in strip cropping using a cnn vegetables detection-characterization method. Comput. Electron. Agric. 193, 106684. 10.1016/j.compag.2022.106684

[B37] Wang Z. Jin L. Wang S. Xu H. (2022). Apple stem/calyx real-time recognition using yolo-v5 algorithm for fruit automatic loading system. Postharvest Biol. Technol. 185, 111808. 10.1016/j.postharvbio.2021.111808

[B38] Wang Z. Zheng L. Liu Y. Li Y. Wang S. (2020). Towards real-time multi-object tracking, in 2020 European Conference on Computer Vision (Glasgow), 107–122.

[B39] Wojke N. Bewley A. Paulus D. (2017). Simple online and realtime tracking with a deep association metric, in 2017 IEEE International Conference on Image Processing (ICIP 2017) (Beijing: IEEE), 3645–3649.

[B40] Wu J. Cao J. Song L. Wang Y. Yang M. Yuan J. (2021). Track to detect and segment: an online multi-object tracker, in 2021 IEEE/CVF Conference on Computer Vision and Pattern Recognition (CVPR 2021), 12347–12356.

[B41] You J. Liu W. Lee J. (2020). A dnn-based semantic segmentation for detecting weed and crop. Comput. Electron. Agric. 178, 105750. 10.1016/j.compag.2020.105750

[B42] Zhang Y. Sun P. Jiang Y. Yu D. Yuan Z. Luo P.. (2021a). Bytetrack: multi-object tracking by associating every detection box. ArXiv, abs/2110.06864. 10.48550/arXiv.2110.06864

[B43] Zhang Y. Wang C. Wang X. Zeng W. Liu W. (2021b). Fairmot: on the fairness of detection and re-identification in multiple object tracking. Int. J. Comput. Vis. 129, 3069–3087. 10.1007/s11263-021-01513-4

[B44] Zhao J. Zhang X. Yan J. Qiu X. Yao X. Tian Y.. (2021). A wheat spike detection method in uav images based on improved yolov5. Remote Sens. 13, 3095. 10.3390/rs13163095

[B45] Zhou X. Koltun V. Krähenbühl P. (2020). Tracking objects as points, in 2020 European Conference on Computer Vision (ECCV 2020) (Glasgow), 474–490.

[B46] Zhou X. Wang D. Krähenbühl P. (2019). Objects as points. ArXiv, abs/1904.07850. 10.48550/arXiv.1904.07850

